# From Benign Polyp to High-Grade Endometrial Sarcoma: A Case Report with Imaging Correlation

**DOI:** 10.3390/diagnostics15172164

**Published:** 2025-08-26

**Authors:** Marina de Miguel Blanc, Cristina Espada González, Milagros Gálvez Montes, Carmen Simón Bejarano

**Affiliations:** 1 Gynecology and Obstetrics Department, Regional Hospital of Axarquía, 29700 Málaga, Spain; 2 Gynecology and Obstetrics Department, Regional University Hospital, 29011 Málaga, Spain; 3 Radiodiagnostic Department, Regional Hospital of Axarquía, 29700 Málaga, Spain

**Keywords:** uterine adenosarcoma, endometrial sarcoma, endometrial cancer, giant polyp, ultrasound imaging, magnetic resonance imaging, hysteroscopy, gynecologic imaging, sarcomatous degeneration, postmenopause bleeding

## Abstract

Uterine adenosarcoma with sarcomatous overgrowth (ASSO) is an exceptionally rare and aggressive subtype of uterine sarcomas, characterized by high mitotic activity, deep myometrial invasion, and an elevated risk of recurrence and metastasis. We report the case of a 79-year-old institutionalized woman with a history of hypertension, type 2 diabetes, chronic hepatitis B, and mild Alzheimer’s disease. During routine hepatic ultrasound surveillance, an incidental 26 mm endometrial lesion was detected. Initial diagnostic hysteroscopy revealed a benign endometrial polyp. However, due to the patient’s institutionalization and absence of gynecologic symptoms, no specialized follow-up was conducted. Four years later, she presented with profuse postmenopausal bleeding. Imaging revealed a markedly enlarged uterus with a 12–13 cm heterogeneous endometrial mass containing cystic and hemorrhagic areas, demonstrating diffusion restriction and significant contrast enhancement on MRI, with no radiologically suspicious lymphadenopathy. Hysteroscopy demonstrated a giant polyp with a broad implantation base; histology suggested sarcomatous transformation. Definitive diagnosis after total hysterectomy with bilateral salpingo-oophorectomy confirmed high-grade ASSO with homologous sarcomatoid overgrowth, consistent with endometrial stromal sarcoma. This case illustrates the progressive malignant transformation of an initially benign-appearing lesion in a patient with significant comorbidities and limited follow-up. It underscores the importance of clinical vigilance, regular monitoring, and interdisciplinary coordination in the evaluation of uterine enlargement in asymptomatic postmenopausal women.

## 1. Background

Uterine sarcomas constitute a heterogeneous and infrequent group of malignant neoplasms of mesenchymal origin, representing approximately 1% of all female genital tract malignancies and 3–7% of uterine corpus cancers [1,2,3,4,5,6,7,8]. These tumors are classified according to their site of origin into three main categories: leiomyosarcomas, arising from myometrial smooth muscle; endometrial stromal sarcomas or undifferentiated sarcomas, originating from endometrial stromal tissue; and mixed epithelial–mesenchymal tumors, such as adenosarcomas [9].

Uterine adenosarcoma is an uncommon subtype, accounting for less than 10% of all uterine sarcomas [10]. Histologically, it is characterized by a biphasic architecture, consisting of benign or mildly atypical glandular epithelium associated with a malignant mesenchymal component, most frequently resembling low-grade endometrial stromal sarcoma. The morphological heterogeneity inherent to uterine sarcomas has historically complicated their classification; however, the 2018 FIGO staging system has refined diagnostic categorization, clearly differentiating adenosarcomas from other uterine sarcomas and establishing four stages based on depth of myometrial invasion, extrauterine spread, and distant metastasis [1].

Within this spectrum, adenosarcoma with sarcomatous overgrowth (ASSO) represents a rare but clinically significant variant, defined by the presence of pure high-grade sarcoma constituting at least 25% of the tumor volume [3,11,12]. The sarcomatous component typically exhibits marked nuclear atypia, high mitotic activity, and deep myometrial invasion, with a strong propensity for lymphovascular dissemination and distant metastases, resulting in a markedly worse prognosis compared with conventional adenosarcoma [13,14]. From a histopathological perspective, the sarcomatous overgrowth may be homologous (comprising native mesenchymal elements such as fibroblasts or smooth muscle cells) or heterologous (containing cartilage, bone, or skeletal muscle tissue). The coexistence of sarcomatous overgrowth with heterologous elements is associated with particularly unfavorable clinical outcomes [15,16].

Diagnosis of ASSO is challenging, as the clinical and radiological features often overlap with benign uterine lesions, such as endometrial polyps or submucosal leiomyomas, particularly in postmenopausal women [5,17]. The condition is frequently asymptomatic in its early stages, and abnormal uterine bleeding—when present—tends to be mild or intermittent, leading to diagnostic delays. In many instances, the lesion is detected incidentally during imaging studies performed for unrelated conditions. Without adequate follow-up, these apparently indolent lesions may progress to large, aggressive tumors with advanced histologic features [18].

From a therapeutic perspective, the standard treatment consists of total hysterectomy with or without bilateral salpingo-oophorectomy. The role of lymphadenectomy, adjuvant radiotherapy, and systemic chemotherapy remains controversial and is typically determined on a case-by-case basis, considering tumor stage, grade, patient comorbidities, and life expectancy. Prognostic factors include tumor size, depth of myometrial invasion, mitotic index, the presence of sarcomatous overgrowth, and the identification of heterologous elements.

The case presented herein documents the progression of an initially benign-appearing endometrial lesion to a high-grade ASSO over several years in an octogenarian patient with multiple comorbidities and incomplete gynecologic follow-up. This case is clinically relevant for its rarity, for the availability of sequential radiological and clinical documentation, and for the absence of gynecologic symptoms during most of the disease course. It highlights the necessity for systematic evaluation of any progressive uterine enlargement in postmenopausal women, regardless of symptomatology, and underscores the importance of interdisciplinary collaboration, involving gynecology, radiology, pathology, geriatrics, and oncology, to ensure timely diagnosis and appropriate management [6,19].

## 2. Case Presentation

At the time of initial diagnosis, a 79-year-old woman had a significant medical history, including long-standing hypertension, type 2 diabetes mellitus managed with oral hypoglycemic agents, chronic hepatitis B virus (HBV) infection under long-term antiviral therapy with tenofovir, and mild Alzheimer’s disease causing cognitive impairment but preserved basic functional autonomy. She was living in a long-term institutional care facility, which, while ensuring consistent primary care, imposed limitations on access to specialized gynecological assessment and routine advanced imaging, particularly when symptoms were absent or nonspecific.

**Figure 1:**
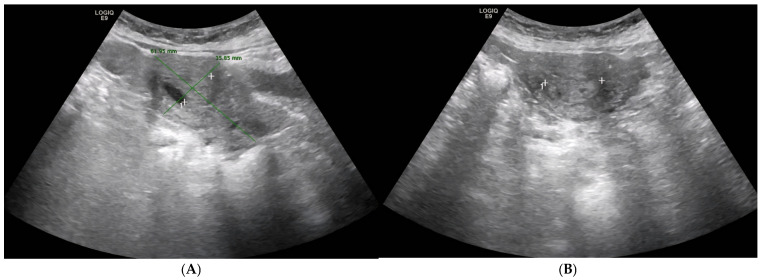
((**A**): Sagittal section view of the uterus, (**B**): Axial section view of the uterus) Abdominal ultrasound (29 July 2021). Uterus measuring approximately 61 × 35 mm. Notably, the endometrial cavity appears dilated and contains a well-defined 17 × 26 mm solid-cystic lesion, prompting a recommendation for gynecologic assessment to exclude neoplasm.

As part of her annual hepatology surveillance protocol for chronic HBV infection, the patient underwent a routine abdominal ultrasound. This examination incidentally revealed distension of the endometrial cavity containing a well-circumscribed intrauterine lesion measuring approximately 26 mm in greatest diameter (see Figure 1A,B). She was referred to gynecology at that time for further evaluation. Diagnostic hysteroscopy was performed, confirming the presence of an endometrial polypoid lesion occupying part of the uterine cavity. The polyp was completely resected under direct visualization without complications. Histopathological examination demonstrated a benign endometrial polyp, with no evidence of hyperplasia, atypia, or malignancy. A retrospective review of the archived report and procedural notes revealed no unusual morphological or technical findings—such as increased stromal cellularity, periglandular thickening, atypical mitotic activity, suspicious necrosis, or a distinctly broad-based implantation—that would have warranted early concern for malignant potential or the need for closer surveillance than is standard for a benign polyp in a postmenopausal patient.

**Figure 2:**
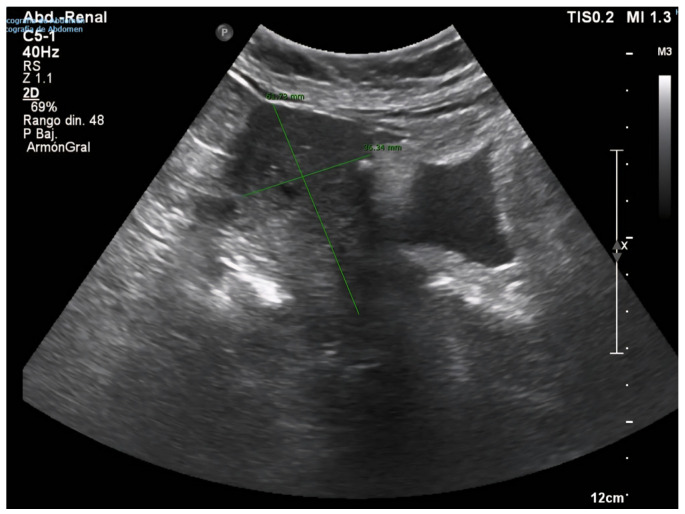
Abdominal ultrasound (7 February 2022). The uterus, measuring approximately 61 × 36 mm, shows calcified myometrial areas compatible with fibroids and persistent endometrial cavity occupation, for which clinical correlation is advised to characterize the lesion.

**Figure 3:**
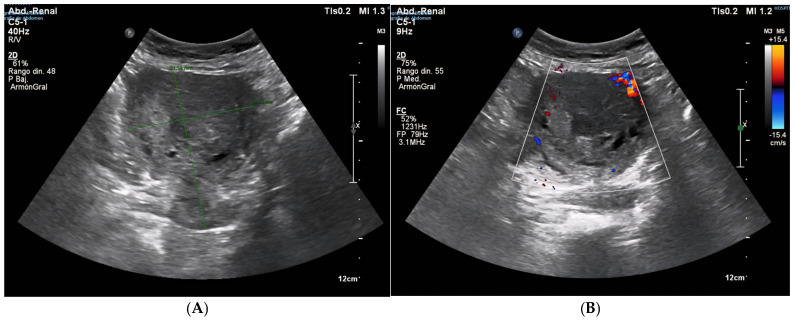
((**A**) Sagittal section view of the uterus, (**B**) Doppler colour exam) Abdominal ultrasound (22 June 2023). The uterus, measuring approximately 81 × 66 mm, shows progression in the appearance of endometrial cavity occupancy, now more evident than on previous imaging, suggesting growth of intracavitary pathology. Highly heterogeneous myometrium-endometrium lacking Doppler flow.

**Figure 4:**
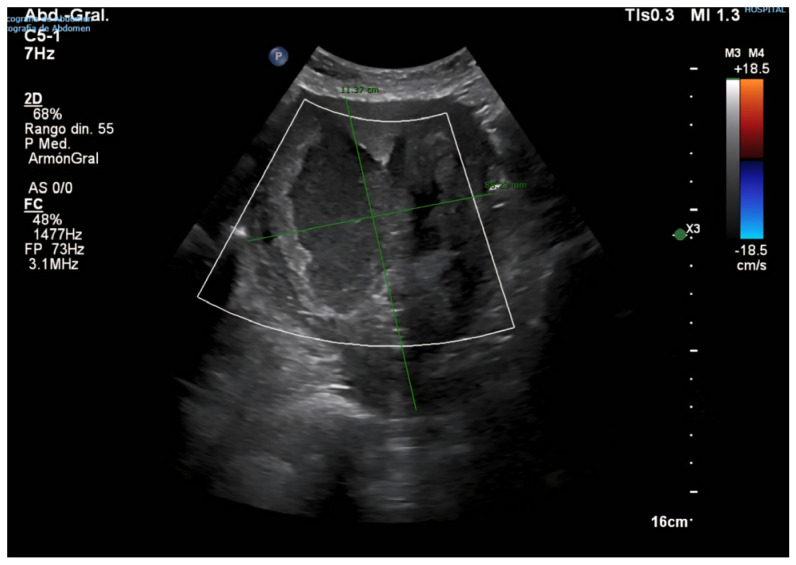
Abdominal ultrasound (23 October 2024). Marked uterine enlargement with highly heterogeneous echotexture. A hypoechoic area, measuring approximately 84 × 13 mm, is identified, eccentric to the endometrial midline and lacking Doppler flow, raising suspicion of a fluid collection or other complex intracavitary mass. Further gynecologic evaluation is advised.

Following this intervention, the patient remained in institutional care, with no reported gynecological symptoms, such as postmenopausal bleeding, pelvic pain, or abnormal discharge. However, progressive uterine enlargement was intermittently noted on annual abdominal ultrasound examinations performed for hepatology follow-up over the subsequent four years (see Figure 2, Figure 3A,B and Figure 4). These findings were not accompanied by targeted pelvic imaging or gynecologic consultation, as the absence of acute or alarming symptoms led to a conservative observational approach by the care team. Consequently, no further endometrial sampling, hysteroscopic assessment, or cross-sectional pelvic imaging was undertaken during this period, and the progressive increase in uterine size was not systematically investigated until the onset of more overt clinical manifestations [20,21].

**Figure 5:**
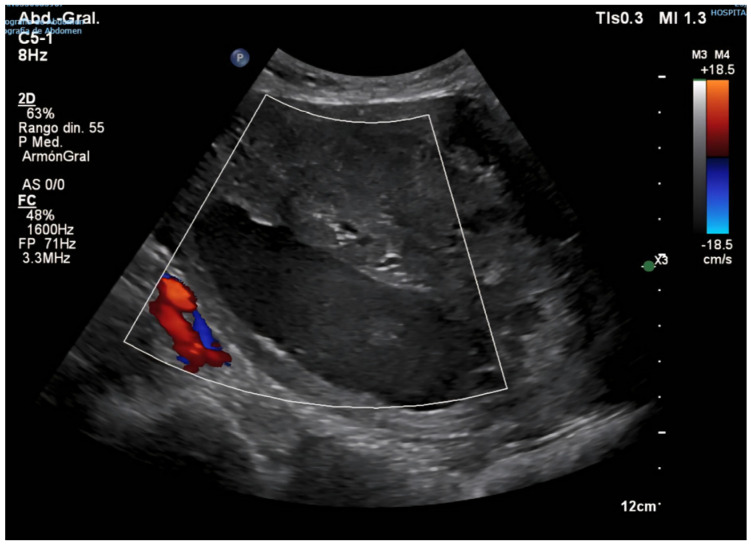
Transvaginal and abdominal ultrasound (16 December 2024). Ultrasound reveals a markedly enlarged uterus entirely occupied by a 10 × 8 cm mixed-echogenic, heterogeneous mass. The myometrial interface is not clearly defined. The absence of a Doppler signal suggests possibilities including a giant endometrial polyp, intracavitary blood clots, or endometrial carcinoma, despite atypical vascular findings.

Four years after the initial gynecologic evaluation, during which no malignant features had been identified, the patient presented with an acute episode of profuse and recurrent postmenopausal bleeding, prompting urgent reassessment. Gynecologic examination revealed a markedly enlarged uterus. Transvaginal ultrasound demonstrated a heterogeneous mixed echogenic uterine mass measuring 10 × 8 cm, with no Doppler evidence of active vascularization, a finding that complicated the diagnostic impression and did not align with the expected vascular pattern of some malignant tumors (see Figure 5).

Given these sonographic findings, the initial differential diagnosis included a large endometrial polyp, intracavitary hematoma, or endometrial carcinoma, despite the lack of Doppler uptake and prior benign pathology. Endometrial biopsy was attempted, but the procedure was inconclusive, as the sample contained predominantly blood clots and scant fragments of atrophic endometrium, insufficient for definitive histological evaluation. Magnetic resonance imaging (MRI) was ordered to further characterize the lesion, assess myometrial and possible extrauterine involvement, and guide subsequent management decisions.

**Figure 6:**
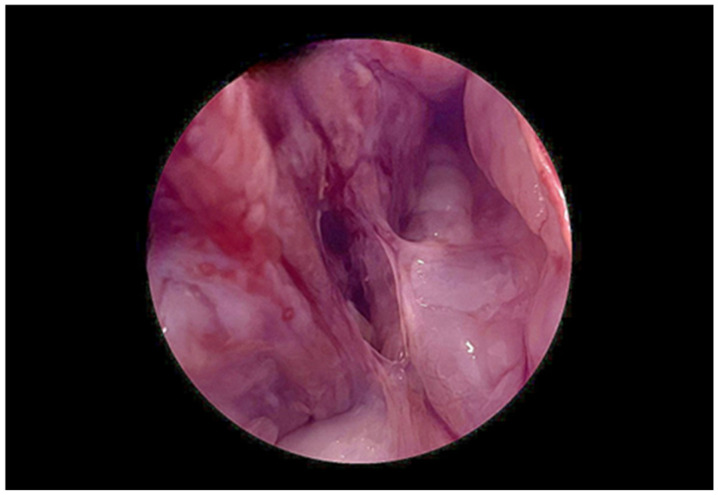
Hysteroscopic view during resectoscopy. A giant endometrial polyp was identified, apparently implanted on the anterior wall. Simultaneous transabdominal ultrasound confirmed an approximately 8 cm intracavitary mass with poor delineation of the anterior myometrial–endometrial interface. The majority of the polyp’s surface appeared lined by normal epithelium, although complete visualization was limited. The posterior endometrial surface appeared diffusely atrophic, except for a whitish, irregular, vascularized area in the left posterolateral region suggestive of atypia. Tubal ostia were not identified. Multiple targeted biopsies were obtained with the resectoscope, prioritizing sampling of the most vascularized area. The procedure was associated with moderate intraoperative bleeding.

While awaiting definitive characterization of the lesion by pelvic MRI, a diagnostic hysteroscopic evaluation under general anesthesia was undertaken to improve visualization and enable targeted tissue sampling. A giant endometrial polyp measuring approximately 8 cm in maximal diameter was identified, apparently implanted on the anterior wall and uterine fundus (see Figure 6). Concurrent transabdominal ultrasound confirmed an intracavitary mass with poor delineation of the endometrial-myometrial interface. Most of the polyp surface appeared covered by normal epithelium; however, the ability to visualize all surfaces in detail was limited by the size of the lesion and the distortion of the uterine cavity. The posterior endometrial surface was diffusely atrophic except for a whitish, irregular, and vascularized area in the left posterolateral region, which was considered highly suspicious for atypical or neoplastic transformation. Tubal ostia were not visualized. Multiple directed biopsies were obtained using a resectoscope, prioritizing the vascularized area. The procedure was complicated by moderate intraoperative bleeding, which was successfully managed with conservative hemostatic measures. Histopathology reported an endometrial polyp with features suggestive of sarcomatous degeneration, warranting further surgical intervention for complete excision and staging.

**Figure 7:**
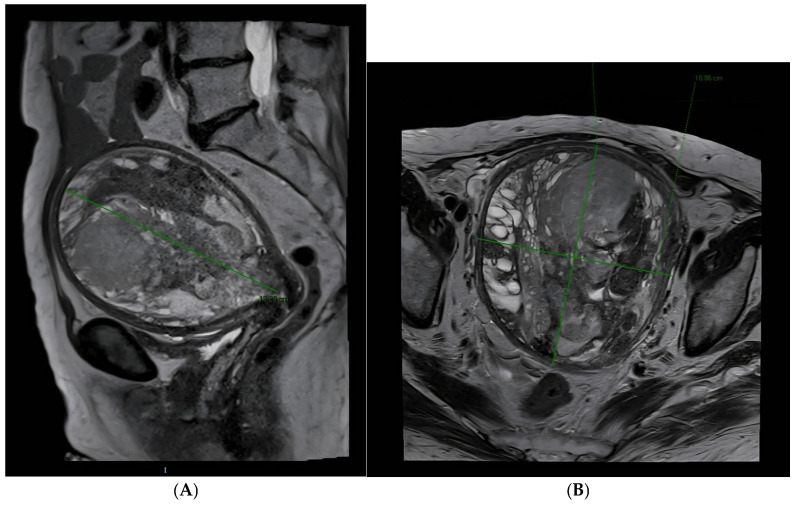
((**A**): Sagittal section view, (**B**): Axial section view) Pelvic Magnetic Resonance Imaging with intravenous contrast (19 February 2025). High-field 1.5 T. MRI demonstrates a markedly enlarged uterus in anteversion, with the endometrial cavity completely filled by a ~12.2 × 10.9 × 13.4 cm heterogeneous mass containing cystic and hemorrhagic areas. No clear implantation base or transition zone is visible, particularly posterolaterally. The lesion shows diffusion restriction and strong post-contrast enhancement, features concerning for a sarcomatous lesion or sarcomatous degeneration of a pre-existing mass.

High-field (1.5 T) pelvic MRI with T2 axial, coronal, and sagittal sequences and contrast administration showed a markedly enlarged anteverted uterus with complete endometrial cavity occupation by a heterogeneous mass (12.2 × 10.9 × 13.4 cm) containing cystic and hemorrhagic areas (see Figure 7A,B). No clear base of implantation was identified, nor was the endometrial-myometrial transition clearly delineated, particularly along the left posterolateral side. The lesion showed restricted diffusion and marked contrast enhancement, extending to the internal cervical os. No significant lymphadenopathy was observed. Cervix, vagina, and adjacent organs appeared unremarkable. A small amount of free fluid was present. Visualized osseous structures were normal [22].

The imaging findings were not consistent with typical endometrioid carcinoma and raised suspicion of a sarcomatous lesion or sarcomatous transformation of a prior benign entity.

The patient consented to surgical management. Intraoperative findings included a markedly enlarged (~15 cm), smooth, and soft uterus. Omentum and bowel appeared macroscopically normal. Ascitic fluid was sampled and tested negative for malignancy. A total abdominal hysterectomy with bilateral salpingo-oophorectomy was performed without complications.

**Figure 8:**
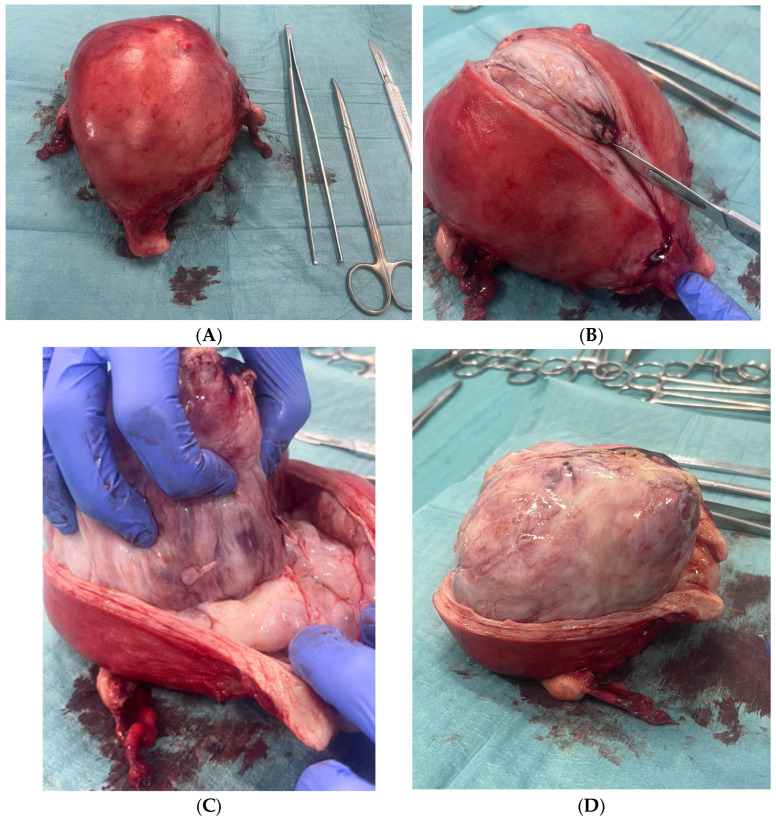
((**A**): Surgical specimen. (**B**–**D**): Dissection of the specimen, cutting at the myometrial level) Macroscopic examination of the total hysterectomy. Specimen, received opened, shows a markedly enlarged uterus measuring 13.5 × 13 × 9 cm. The endometrial cavity is completely occupied by a large intracavitary mass measuring approximately 10 cm in diameter, with gross features suggestive of sarcomatous transformation. The mass displays a lobulated, heterogeneous surface with areas of hemorrhage and necrosis. The cut surface reveals alternating solid and cystic regions composed of tan-white fleshy tissue interspersed with hemorrhagic zones, consistent with a giant endometrial polyp showing features suspicious for sarcomatous degeneration. No obvious myometrial invasion is identified on gross inspection. The left adnexa includes an ovary measuring 2 cm and a fallopian tube of 5 cm; the right adnexa similarly consists of a 2 cm ovary and a 5 cm fallopian tube, both without gross abnormalities.

Macroscopic examination revealed a fleshy, multinodular, pearly-surfaced polypoid mass with extensive internal hemorrhagic areas (see Figure 8A–D).

**Figure 9:**
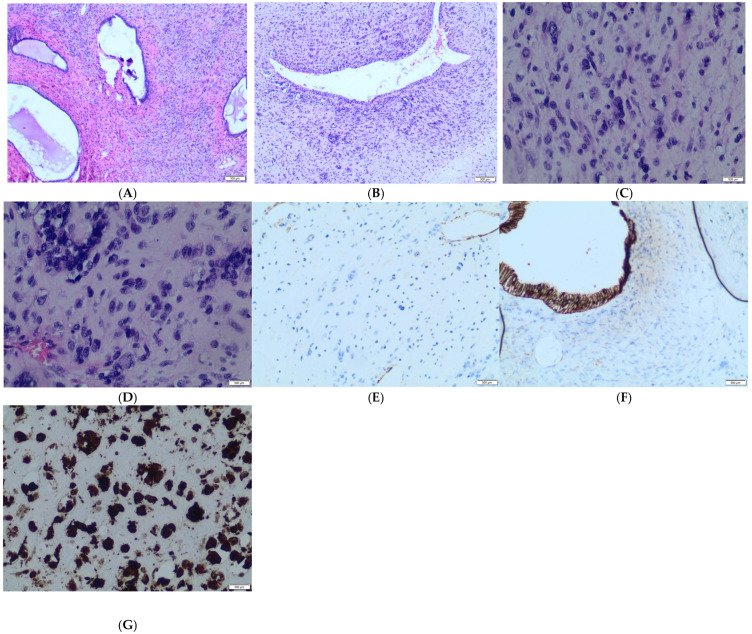
(**A**) Hematoxylin and Eosin (40×): Sarcomatous areas and benign epithelial component. (**B**) Hematoxylin and Eosin (60×): Sarcomatous area. (**C**) Hematoxylin and Eosin (60×): Sarcomatous area. (**D**) Same as Figure 1. (**E**) Vimentin (60×): Positive staining in sarcomatous area. (**F**) Broad-spectrum cytokeratins: Positive staining in the benign epithelial component and negative staining in the sarcomatous component. (**G**) Sarcomatous area negative for cytokeratins.

The Final histopathological diagnosis was high-grade uterine adenosarcoma with homologous sarcomatous overgrowth consistent with endometrial stromal sarcoma (See Figure 9A–G), exhibiting pleomorphic multinucleated cells and up to 15 mitoses per 10 high-power fields. Immunohistochemistry was positive for vimentin, CD10, cyclin D1, p16, and WT1, with weak/focal PR and actin positivity, and wild-type p53 expression. Ovaries were atrophic and free of tumor infiltration.

The patient’s case was subsequently presented for discussion at a multidisciplinary tumor board, comprising specialists in gynecologic oncology, radiology, pathology, anesthesiology, geriatrics, and palliative care. This collaborative review took into account not only the definitive histopathologic diagnosis and the aggressive biological potential of the tumor but also the patient’s advanced chronological age, institutionalized status, baseline functional limitations, and significant comorbidities, including hypertension, type 2 diabetes mellitus, chronic hepatitis B infection under antiviral therapy, and mild Alzheimer’s disease. After weighing the anticipated benefits of adjuvant oncologic therapy against the substantial risks of treatment-related morbidity and the limited likelihood of meaningful improvement in survival outcomes, the board reached a consensus recommendation to refrain from pursuing active oncologic interventions such as chemotherapy or radiotherapy.

The postoperative course was largely uneventful, with the patient recovering without major complications and regaining her baseline functional status. In alignment with the tumor board’s conclusions and following detailed discussions with the patient’s legal representatives and close family members, an informed decision was made to proceed with a strategy of close symptomatic clinical surveillance. This approach prioritized comfort, preservation of quality of life, and avoidance of potentially burdensome treatments, while maintaining vigilance for any signs of local recurrence or disease progression. No adjuvant therapy was initiated, and the follow-up plan included periodic gynecologic assessments and symptom monitoring within the institutional care setting.

## 3. Discussion

Uterine adenosarcoma with sarcomatous overgrowth (ASSO) is a rare but clinically significant malignancy due to its aggressive course. Its prevalence among uterine adenosarcomas varies across series but consistently portends a significantly worse prognosis compared to adenosarcomas without overgrowth [3,23].

Unlike classic adenosarcomas, which typically present with well-differentiated stroma, low mitotic activity, and normal TP53 expression, ASSO is marked by aggressive features. These include tumor size > 10 cm, necrosis, hemorrhage, infiltrative growth pattern, and frequent myometrial and lymphovascular invasion [13,24]. Histologically, ASSO exhibits poorly differentiated stroma, pleomorphic cells, and a high mitotic rate (10–22 mitoses per 10 HPF). Aberrant TP53 expression is common and is associated with higher risks of recurrence and metastasis.

Recent studies confirm that adverse outcomes in uterine adenosarcoma, particularly with sarcomatous overgrowth, are associated with large tumor size, deep myometrial and lymphovascular invasion, older age, and advanced FIGO stage. High-grade tumors often carry TP53 pathway alterations, while low-grade forms usually retain wild-type expression. Other recurrent molecular changes include DICER1, ARID1A, FGFR2, and MDM2/CDK4 amplifications, suggesting genetic heterogeneity and potential therapeutic targets in selected cases [25].

The co-presence of sarcomatous overgrowth and heterologous elements significantly increases the risk of local recurrence, metastasis, and mortality [14,26]. This case is particularly illustrative, documenting the evolution from an incidentally detected benign endometrial lesion (26 mm) to a massive 13–15 cm uterine tumor with overt malignant characteristics, ultimately diagnosed as high-grade ASSO after hysterectomy. The progression occurred in an elderly institutionalized patient without gynecological symptoms for over four years, leading to a critical delay in specialized care [5,20].

Ultrasound typically shows a polypoid endometrial mass but can be limited by necrosis, hemorrhage, or poor Doppler flow. MRI offers superior characterization, with adenosarcomas often appearing as intracavitary polypoid masses with cystic or hemorrhagic areas, helping distinguish them from endometrioid carcinoma or leiomyosarcoma. Current consensus supports contrast-enhanced MRI for risk assessment and surgical planning when sarcoma is suspected [27].

This outcome highlights a gap in the management of incidental gynecologic findings in postmenopausal women, particularly when follow-up is hindered by factors such as institutionalization, cognitive decline, or asymptomatic presentation [28,29].

International guidelines advocate systematic evaluation of any progressive uterine enlargement in postmenopausal women, even in the absence of bleeding, through regular ultrasound surveillance, advanced imaging modalities (e.g., pelvic MRI), and hysteroscopic biopsy of persistent or suspicious lesions [1,6,30].

In this case, the four-year follow-up delay was largely due to institutionalization and the absence of symptoms. It underscores the necessity of multidisciplinary coordination to ensure the execution of recommended evaluations, even in asymptomatic patients [6,19].

No strong hereditary syndromes have been linked to uterine adenosarcoma, but alterations in TP53 and DNA repair–related genes, such as ATM, may guide individualized therapy in advanced or recurrent disease. Germline testing is not routine, though selective molecular profiling may be valuable, particularly in high-grade cases [31].

In elderly institutionalized women, decision-making must balance oncologic thoroughness with quality of life. Nonetheless, the silent progression of seemingly benign lesions—as in this case—warrants sustained clinical vigilance and follow-up, especially for persistent or enlarging endometrial masses [6,9,19].

## 4. Conclusions

This case provides a detailed and sequential account of the transformation of an initially benign endometrial lesion into high-grade uterine adenosarcoma with sarcomatous overgrowth (ASSO), emphasizing the rarity and the clinical relevance of this aggressive tumor [6,13].

It highlights the importance of maintaining high clinical suspicion in cases of postmenopausal bleeding or unexplained uterine enlargement, even in asymptomatic patients. Active surveillance of incidental gynecological findings in routine assessments of postmenopausal women is essential, particularly in institutionalized patients or those with fragmented medical care [1,27]. The execution of interconsultation recommendations among primary care physicians, radiologists, geriatricians, internists, and gynecologists must be ensured to prevent diagnostic delays caused by discontinuity of care [6,19,30].

Structured multidisciplinary collaboration is crucial to enable early diagnosis, accurate interpretation of clinical and imaging findings, and the design of personalized treatment strategies that balance oncological efficacy with patient quality of life [19,24,28].

Ultimately, this case underscores the vital role of proactive surveillance and collaborative decision-making in managing rare but high-risk conditions such as ASSO, whose prognosis can improve significantly with early detection and a comprehensive care approach [3,14,25].

## Data Availability

The data are available upon request from the authors.
